# Noni (*Morinda citrifolia*) Modulates the Hypothalamic Expression of Stress- and Metabolic-Related Genes in Broilers Exposed to Acute Heat Stress

**DOI:** 10.3389/fgene.2017.00192

**Published:** 2017-12-05

**Authors:** Hossein Rajaei-Sharifabadi, Laura Ellestad, Tom Porter, Annie Donoghue, Walter G. Bottje, Sami Dridi

**Affiliations:** ^1^Center of Excellence for Poultry Science, University of Arkansas, Fayetteville, AR, United States; ^2^Department of Animal and Avian Sciences, University of Maryland, College Park, MD, AR, United States; ^3^Poultry Production and Product Safety Research Unit, United States Department of Agriculture, Agricultural Research Service, Fayetteville, AR, United States

**Keywords:** acute heat stress, AMPK-mTOR pathway, broiler, hypothalamus, Noni

## Abstract

Heat stress (HS) adversely affects growth performance and inflicts heavy economic losses to the poultry industry. There is, therefore, a critical need to identify new alternative strategies to alleviate the negative effects induced by HS. The tropic medicinal plant, *Morinda citrifolia* (Noni), is being used in livestock nutrition, however the literature is limited and conflicting for its impact on growth performance. The present study aimed to determine the effect of Noni on feeding and drinking behavior as well as on the hypothalamic expression of stress- and metabolic-related genes in broiler chickens exposed to acute HS. A total of 480 1 day-old male broiler chicks were randomly assigned to 12 controlled environmental chambers. Birds were subjected to two environmental conditions (TN, 25°C vs. HS, 35°C for 2 h) and fed two diets (control vs. 0.2% Noni) in a 2 × 2 factorial design. Feed intake and core body temperature (BT) were recorded during HS period. Blood was collected and hypothalamic tissues were harvested for target gene and protein analyses. Acute HS-broilers exhibited higher BT (~1°C), spent less time eating with a significant decrease in feed intake, and spent more time drinking along with higher drinking frequency compared to those maintained under TN conditions. Although Noni supplementation did not improve feed intake, it significantly delayed (~30 min) and reduced the BT-induced by HS. At molecular levels and under HS conditions, Noni supplementation down regulated the hypothalamic expression of HSP90 and its related transcription factors HSF1, 2, and 4, increased orexin mRNA levels, and decreased the phosphorylation levels of AMPKα1/2^Thr172^ and mTOR^Ser2481^. Together, these data indicated that Noni supplementation might modulate HS response in broilers through central orexin-AMPK-mTOR pathways.

## Introduction

Livestock production, including poultry is facing substantial challenges from steep projected increases in global demand for animal proteins due to predicted increases in world human population on one hand, and the need to adapt to extreme environmental conditions due to climate change on the other hand. Indeed, large, abrupt, and widespread extreme heat waves have occurred repeatedly in the past (Alley et al., 2005) and are predicted to increase for the next century (Mora et al., 2013). Environmental HS impacts every aspect of animal lives and their very existence (Chen et al., 2011). It can result in heat-related discomfort, illness, multiple organ damage, and under extreme conditions can cause spiraling hyperthermia leading to death. In broiler chickens, which play a key role in worldwide meat production, the strong negative effects of HS on feed intake, growth, feed efficiency, meat yield, and mortality leading to dramatic economic losses are well-documented (Dale and Fuller, 1980; Cahaner and Leenstra, 1992; Geraert et al., 1996; Deeb and Cahaner, 2002). Such effects will take a heavy toll during the next decades as the distribution of heat anomalies continues to rise.

Emerging evidence indicates that the regulation of energy homeostasis (energy intake and expenditure) and the stress response are tightly coupled physiological processes (Valles et al., 2000). At the cellular levels, HS rapidly initiates the increased synthesis of chaperone proteins belonging to the heat shock protein (HSP) family that play pivotal housekeeping functions and orchestrate folding/unfolding and assembly/disassembly of protein complexes to maintain cellular homeostasis. These proteins are transcriptionally regulated through heat shock factors (HSF) that bind to heat shock response element (HSE) in the upstream promoter regions of HSP (Shuey and Parker, 1986). Depending on the type of insult and the stress amplitude (type, duration, degree, severity), cells can employ the survival pathway via activating protein quality control systems or proceed into cell-death through activation of stress signaling cascades. At the organismal levels, the stress-mediated cellular alterations induces various neuroendocrine, physiological, and immunological adaptation that lead to marked variations in energy homeostasis as well as in intermediary metabolism (Portner, 2004). Energy homeostasis is tightly controlled by complex hypothalamic molecular networks that are still not completely defined, at least in birds.

Although an enormous amount of important work has been done to determine the physiological and behavioral responses to HS and improve management and/or nutritional strategies to alleviate these negative effects, poultry productivity still declines during hot seasons. Furthermore, due to recent concerns regarding the usage of antibiotics and inorganic additives in poultry, research efforts were shifted toward alternative options in the form of organic additives from plant-based compounds and extracts to ameliorate heat stress productivity loss. A large number of phytogenic plants with known medicinal properties are available and are being used by poultry producers. The tropic plant *Morinda citrifolia* L. (Noni), also known as Indian Mulberry or Great Morinda (English), Ashyuka (Sanskrit), Ba Ji Tian (China), Cheese Fruit or Canary Wood (Australia), Hrudi (Bengali), and various other names in different countries, is a popular medicinal plant belonging to the coffee family, Rubiaceae. Noni-derived products are rich in a variety of phytochemicals and antioxidants (Potterat and Hamburger, 2007; Lin et al., 2012) and have been used in human consumption as a health promoter and a growth tonic (McClatchey, 2002; Chang et al., 2013; Lin et al., 2013; Gironés-Vilaplana et al., 2015; Nerurkar et al., 2015). They have been commercialized in the USA since the 1990 s and fruit juice of Noni has been approved as a Novel Food by the European Commission in 2003. Products derived from the fruit and leaves of Noni have also gained considerable popularity in animal nutrition. A large number of beneficial effects have been claimed for Noni, and though many of the reports are only available as congress abstracts and peer-reviewed research papers on these effects are lacking. Giving Noni extract has been reported to increase appetite and improve feed intake, weight gain, and consumption of drinking water in broilers, however its underlying molecular mechanism is still unknown. We hypothesize that Noni supplementation would ameliorate HS productivity loss in broilers and we undertake the present study as a first step to determine the effects of Noni-supplemented diet on feed intake, circulating metabolites and hormones, and on the expression of hypothalamic HSPs and feeding-related (an)orexigenic neuropeptides in acute heat-stressed broiler chickens.

## Materials and methods

### Animals, experimental design, and sampling

The present study was conducted in accordance with the recommendations in the guide for the care and use of laboratory animals of the National Institutes of Health and the protocol was approved by the University of Arkansas Animal Care and Use Committee. A total of 480 1 day-old by-product male chicks (Cobb 500) were weighed (average BW was 45.1 ± 1 g) and randomly assigned to 12 controlled environmental chambers. Each chamber was divided into 2 floor pens (3.6 × 1.4 m) covered with pine wood shaving and equipped with separate feeders and water lines. In each chamber, chicks (20 per pen) were fed corn-soy based diets (Control, C) or the same diet containing 2 g dried noni plant/kg feed (Noni, N) (16.3 MJ ME kg^−1^ and 210 g crude protein kg^−1^). The dose of Noni has been chosen based on the commercial practice in the poultry industry in tropic areas. Birds were given *ad libitum* access to clean water and food. The ambient temperature was reduced gradually from 32 to 25°C at 21 days of age. A relative humidity of ~20% and a 23 h light/1 h dark cycles were also maintained until the end of the experiment. On d 22, birds were weighed and they had similar average body weight of 1,000 ± 42 g. The ambient temperature was increased within 10 min to reach 35°C in eight chambers to induce acute heat stress (HS, 2 h after the temperature reached 35°C) with the remaining four chambers maintained at 25°C (TN). The day before the acute HS, one chicken per pen was randomly selected and equipped with a Thermochron temperature logger (iButton, Embedded Data Systems, KY) for continuous monitoring of core temperature. The environmental temperature and humidity were also continuously recorded in each chamber. After 2 h heat stress, feed intake was determined and the data logger-equipped chickens were humanly killed by cervical dislocation. Blood samples were collected in vacutainer tubes with PST gel and lithium heparin (BD, NJ) and after centrifugation (1,500 g, 10 min, 4°C), plasma was separated and stored at −20°C for later analyses of circulating metabolites and hormones. Hypothalamus samples were dissected as we previously described (Piekarski et al., 2016) and flash frozen on dry ice and kept at −80°C for subsequent gene and protein expression analyses.

### Feeding and drinking behaviors

Feeding and drinking behaviors were recorded simultaneously by two video cameras (Canon XA25 and Sony HD Handycam PJ304) placed in front of feeders and drinkers in each HS and TN chamber. Videotaped recording were watched on a screen monitor at normal and slower speed to distinguish six behavioral states: time spent eating (pecking in the feeders), time spent drinking (pecking in the drinker nipples), feeding frequency (number of visits to feeders), drinking frequency (number of visits to drinkers), time spent lying down, and activity time (difference between total observation time and sum of the times spent feeding, drinking, and lying down). Two consecutive visits to feeders or drinkers with <15 s break were considered as one event.

### Plasma metabolites and hormone measurement

As we previously described (Nguyen et al., 2015), commercial colorimetric diagnostic kits were used to measure plasma glucose (Ciba Corning Diagnostics Corp., OH), triglycerides, cholesterol, and creatine kinase (CK, Chiron Diagnostics, Cergy Pontoise, France), lactate dehydrogenase (LDH, Bayer Healthcare, Dublin, Ireland), and uric acid levels (UA, Pointe Scientific Inc., Canton, MI) with an automated spectrophotometer according to manufacturer's recommendations. Corticosterone was measured with a commercially available ELISA kit (Cat# ADI-900-097, Enzo Life Sciences, Farmingdale, NY), according to manufacturer's instructions. Plasma levels of total 3, 5, 3′-triiodothyronine (T_3_) and thyroxine (T_4_) were measured using coated tube radioimmunoassay kits (MP Biomedicals, Solon, OH), with the following modifications. The sensitivities of both standard curves were extended to 0.03 ng/mL (T_3_) and 1.5 ng/mL (T_4_) by performing a series of 2-fold dilutions with steroid-free serum (MP Biomedicals, Solon, OH), samples were diluted 1:5 (T_3_ only) using steroid-free serum (MP Biomedicals, Solon, OH), and tubes were incubated for 16 h at 4°C following addition of tracer. Radioactivity retained in each tube was counted for 1 min with a gamma counter (Wallac 1470 Wizard Automatic Gamma Counter, Perkin Elmer Life Sciences, Waltham, Massachusetts). Intra-assay coefficient of variation values were 5.1 and 5.9% for T_3_ and T_4_, respectively.

### RNA isolation, reverse transcription, and quantitative real-time PCR

Total RNA was extracted from chicken hypothalamus by Trizol reagent (ThermoFisher Scientific, Rockford, IL) according to manufacturer's recommendations, DNAse treated and reverse transcribed (Quanta Biosciences, Gaithersburg, MD). RNA integrity and quality was assessed using 1% agarose gel electrophoresis and RNA concentrations and purity were determined for each sample by Take three micro volume plate using Synergy HT multi-mode microplate reader (BioTek, Winooski, VT). The RT products (cDNAs) were amplified by real-time quantitative PCR (Applied Biosystems 7500 Real-Time PCR system) with SYBR Green Master Mix (ThermoFisher Scientific, Rockford, IL). Oligonucleotide primers used for chicken hypothalamic neuropeptide Y (NPY, Accession n° NM_205473), agouti-related peptide (AgRP, Accession n° AB029443), proopiomelanocortin (POMC, Accession n° AB019555), cocaine and amphetamine regulated transcript (CART, Accession n° KC249966), orexin (ORX, Accession n° AB056748), orexin receptor 1/2 (ORXR1/2, Accession n° AB110634 and NM_001024584, respectively), leptin receptor (Ob-R, Accession n° NM_204323), HSP 60, 70 (HSP60, 70, Accession n° NM_001012916 and JO2579), heat shock factor 1-4 (HSF1-4, Accession n° L06098, NM_001167764, L06126, and NM_001172374, respectively), AMP-activated protein kinase alpha 1/2 (AMPKα1/2, Accession n° NM_001039603 and NM_001039605, respectively), mechanistic target of rapamycin (mTOR, Accession n° XM_417614), p70 ribosomal protein S6 kinase 1 (S6K1, Accession n° NM_001109771), and ribosomal 18S (Accession n° AF173612) as housekeeping gene were previously published (Blankenship et al., 2015; Lassiter et al., 2015; Nguyen et al., 2015). Oligonucleotide primers used for chicken HSP90 (Accession n° X07265) are: forward, 5′-TGACCTTGTCAACAATCTTGGTACTAT-3′ and reverse, 5′-CCTGCAGTGCTTCCATGAAA-3′ amplifying a fragment of 68 bp. The qPCR cycling conditions were 50°C for 2 min, 95°C for 10 min followed by 40 cycles of a two-step amplification program (95°C for 15 s and 58°C for 1 min). At the end of the amplification, melting curve analysis was applied using the dissociation protocol from the Sequence Detection system to exclude contamination with unspecific PCR products. The PCR products were also confirmed by agarose gel and showed only one specific band of the predicted size. For negative controls, no RT products were used as templates in the qPCR and verified by the absence of gel-detected bands. Relative expressions of target genes were determined by the 2^−ΔΔCt^ method (Schmittgen and Livak, 2008).

### Western blot analysis

Total proteins were extracted from chicken hypothalamus, quantified, and subjected to Western blot as we previously described (Nguyen et al., 2015). The rabbit polyclonal anti-phospho mechanistic target of rapamycin (mTOR)^Ser2481^ (#2971), anti-mTOR (#2972), anti-phospho AMP-activated protein kinase alpha (AMPKα1/2)^Thr172^ (#2531), anti-AMPKα1/2 (#2795), anti-HSP90 (#PA5-17610), goat polyclonal anti-HSP60 (#sc-1052), and mouse monoclonal anti-HSP70 (#MAI-91159) were used. Protein loading was assessed by immunoblotting with the use of rabbit anti-β actin (#4967). Pre-stained molecular weight marker (Precision Plus Protein Dual Color) was used as a standard (BioRad, Hercules, CA). All primary antibodies were purchased from Cell Signaling Technology (Danvers, MA), except for the anti-HSP70 and anti-HSP90 which were purchased from Pierce Thermo Scientific (Rockford, IL) and anti-HSP60 from Santa Cruz Biotechnology (Dallas, TX). The secondary antibodies were used (1:5,000) for 1 h at room temperature. The signal was visualized by enhanced chemiluminescence (ECL plus) (GE Healthcare Bio-Sciences, Buckinghamshire, UK) and captured by FluorChem M MultiFluor System (Proteinsimple, Santa Clara, CA). Image Acquisition and Analysis were performed by AlphaView software (Version 3.4.0, 1993–2011, Proteinsimple, Santa Clara, CA).

### Statistical analysis

Data (growth performances, circulating parameters, and gene/protein expression) were analyzed by two-factor ANOVA with diet (control, C vs. Noni, N) and environmental conditions (TN vs. HS) as classification variables. Data for core body temperature were analyzed by two-way repeated measures ANOVA. If ANOVA revealed significant effects, the means were compared by Tukey multiple range test using the Graph Pad Prism version 6.00 for Windows (Graph Pad Software, La Jolla California, USA). Differences were considered significant at *P* < 0.05.

## Results

### Acute HS increases body temperature and alters feeding and drinking behaviors in broilers

**Figure 1:**
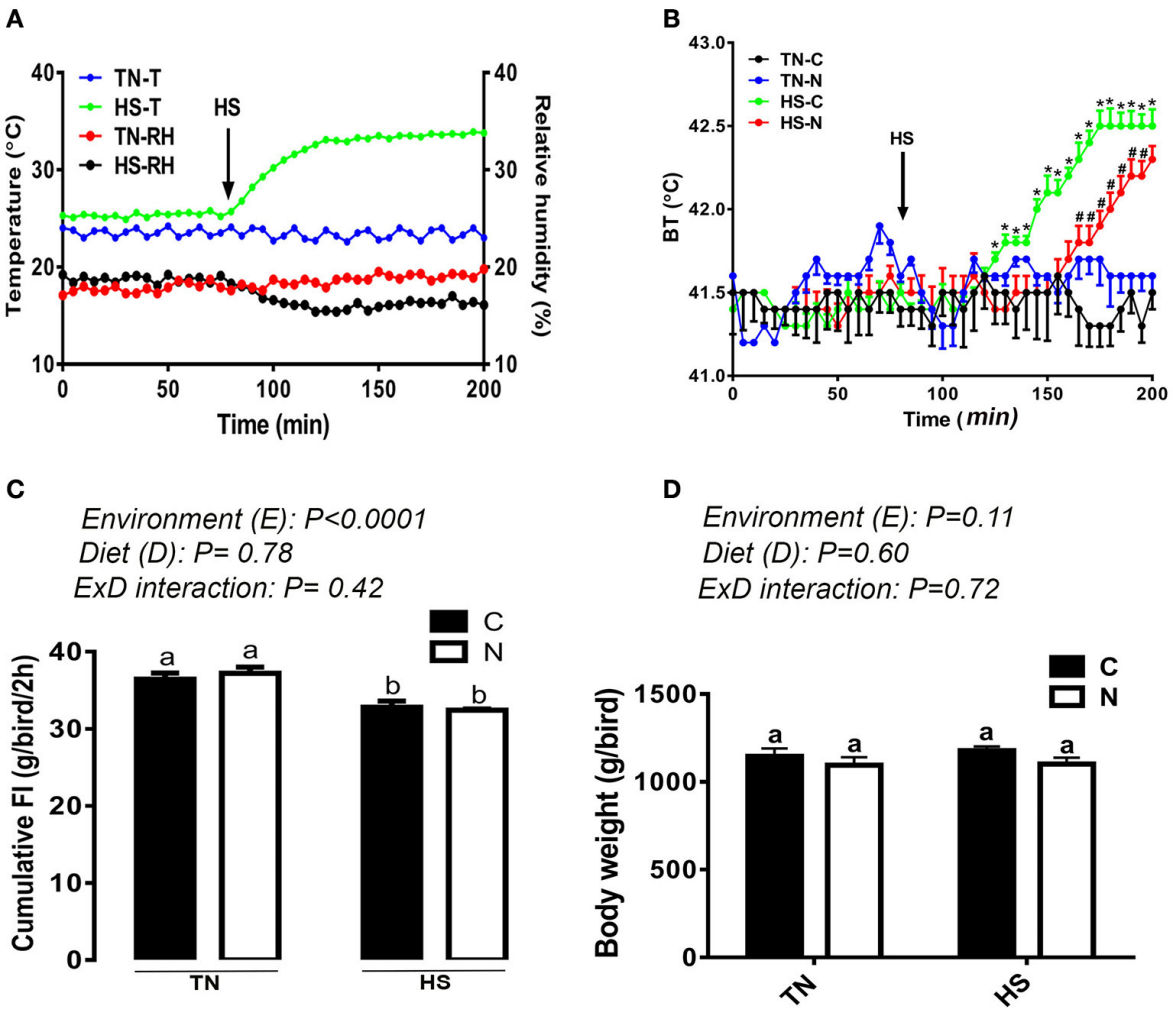
Effects of 2 h-HS exposure on core body temperature, feed intake, and body weight in broiler chickens. Acute HS **(A)** increased core body temperature (BT) **(B)** and decreased feed intake in broiler chickens **(C)** without affecting body weight **(D)**. Data are presented as mean ± SEM (*n* = 8/group for BT and *n* = 160/group for BW and FI). Different letters and different symbols indicate significant difference at *P* < 0.05. TN-T and HS-T, temperature of the room in thermoneutral and heat stress conditions, respectively. TN-RH and HS-RH, relative humidity of the barn in thermoneutral and heat stress conditions, respectively.

**Figure 2:**
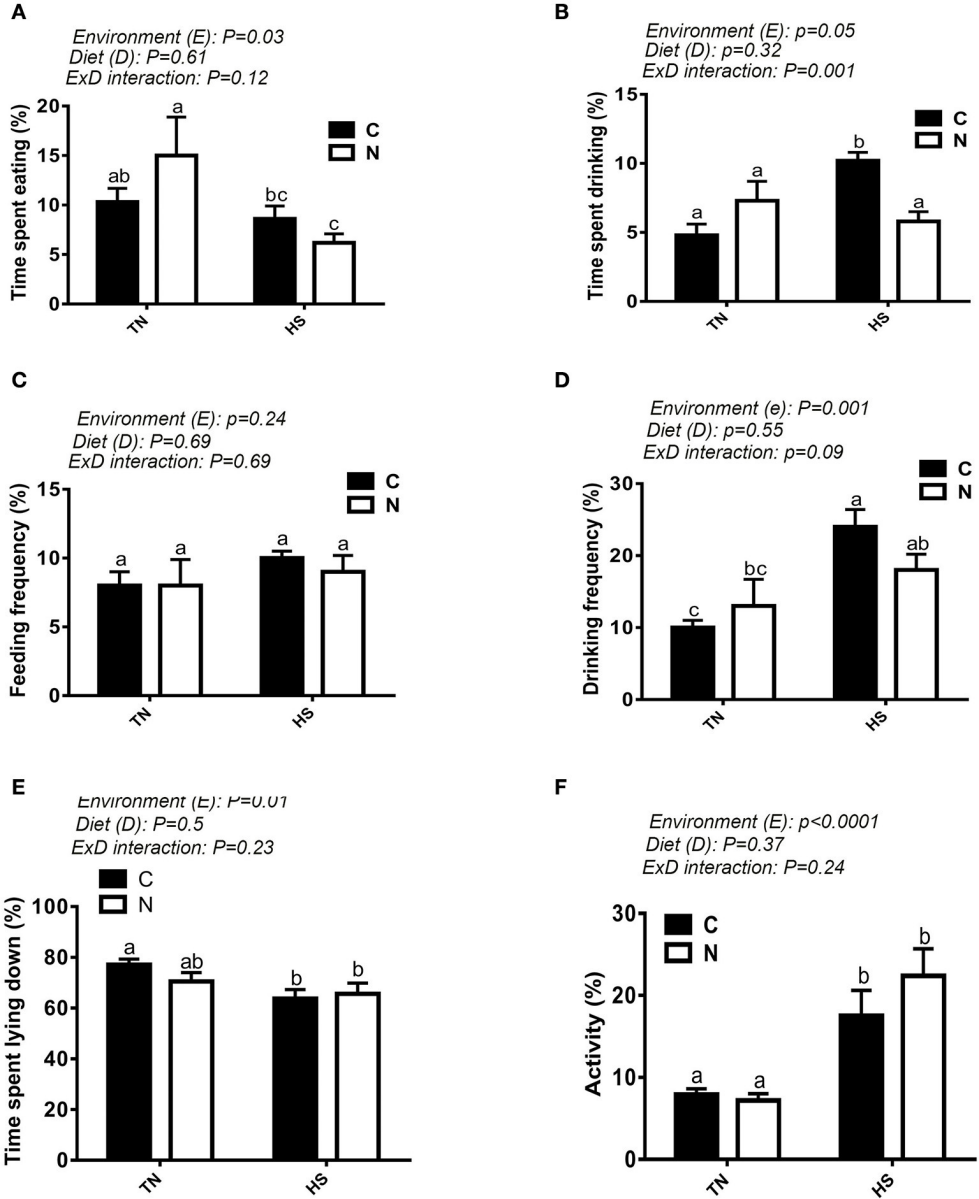
Effect of HS on feeding and drinking behaviors. Time spent eating **(A)**, time spent drinking **(B)**, feeding frequency **(C)**, drinking frequency **(D)**, time spent lying down **(E)**, and activity **(F)** were recorded using video camera. Data are presented as mean ± SEM (*n* = 5/group). Different letters indicate significant difference at *P* < 0.05.

As shown in Figure 1A, a 10°C increase in ambient temperature at a relative humidity of ~20% increased the body temperature (BT) of broiler chickens by ~1°C (Figure 1B). The BT of the control-fed group increased 1 h after the onset of the HS, however it was delayed by ~30 min in the Noni-fed group (Figure 1B). Additionally, Noni supplementation reduced the increased BT-induced by HS. Voluntary feed consumption was negatively affected by acute HS (*P* < 0.05, Figure 1C) in both control and Noni-fed groups, however body weight did not differ between all groups (Figure 1D). Although feeding frequency (number of visits to feeders) did not differ between the experimental groups (Figure 2C), acute heat stressed chickens spent less time eating compared to TN group (Figure 2A) which may explain the reduction of feed intake. Acute HS chickens spent more time drinking along with higher drinking frequency compared to the TN group and this was more pronounced in the control than in the Noni group (Figures 2B,D). Heat-stressed chickens exhibited higher activity and less lying down time compared to the TN group and this was obvious in both control and Noni-fed chickens (Figures 2E,F).

### Acute HS alters circulating hormone and metabolite levels in broilers

**Figure 3:**
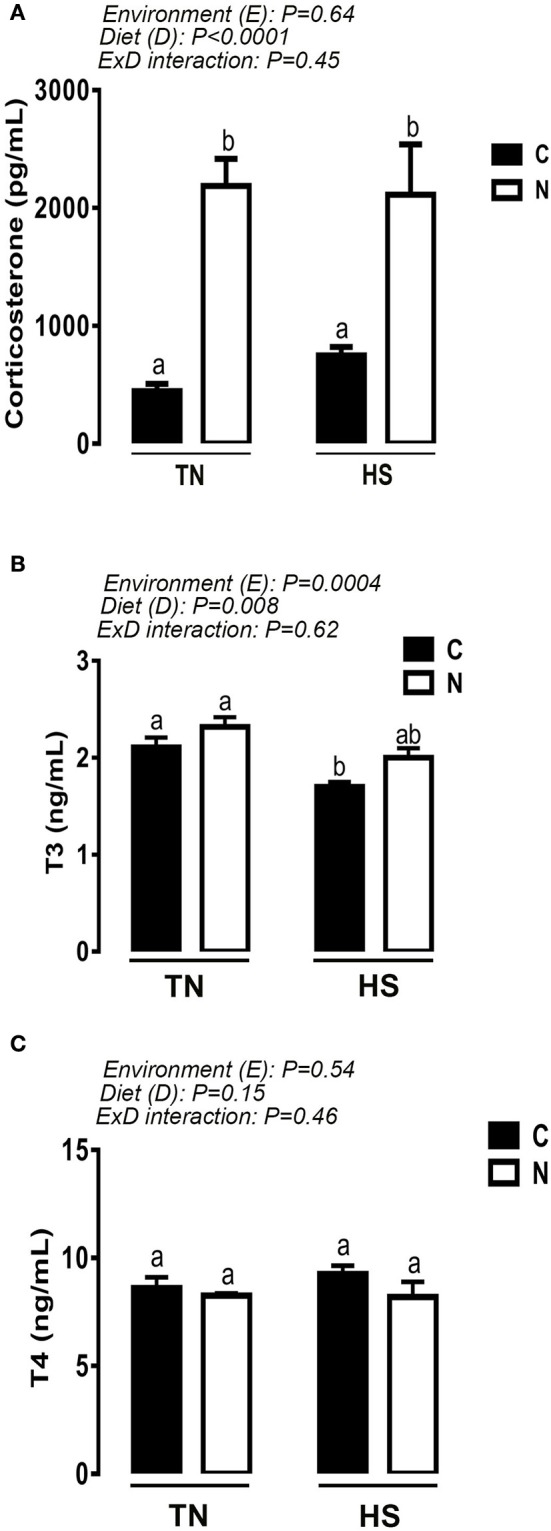
Effect of 2 h-HS exposure on circulating hormone levels. Plasma corticosterone levels were measured using ELISA **(A)**, and circulating T3 **(B)** and T4 **(C)** concentrations were measured using RIA as described in materials and methods. Data are presented as mean ± SEM (*n* = 10/group). Different letters indicate significant difference at *P* < 0.05.

As shown in Table 1, plasma glucose, cholesterol, triglyceride, LDH, and CK levels remained unchanged between control and Noni-fed groups under both TN and acute HS conditions. Interestingly, acute HS significantly decreased the circulating levels of uric acid in both control and Noni-fed chickens (Table 1). Dietary supplementation of Noni significantly increased plasma corticosterone levels in broilers maintained under both TN and HS conditions (Figure 3A). Although acute HS decreased plasma T3 concentrations in broilers fed control diet, Noni-fed chickens showed similar circulating levels of T3 under both HS and TN conditions (Figure 3B). The circulating concentration of T4 was not affected by experimental treatments (Figure 3C).

**Table 1:** Effects of acute HS and Noni on circulating metabolite levels in broiler chickens.

**Metabolites^c^**	**Experimental groups^a^**				***P*–value^b^**		
	**TN**		**HS**				
	**C**	**N**	**C**	**N**	**E**	**D**	**ExD**
*Glc (mg/dL)*	286.5 ± 16	286 ± 12	289.1 ± 7	289 ± 6	0.7	0.9	0.9
*Chol (mg/dL)*	151.5 ± 3	150.7 ± 4	145.5 ± 4	139.1 ± 6	0.1	0.5	0.6
*TG (mg/dL)*	39 ± 5	47.2 ± 5	39 ± 4	40 ± 4	0.4	0.3	0.4
*LDH (U/L)*	37 ± 7	37.5 ± 5	52.7 ± 5	41.3 ± 5	0.1	0.3	0.3
*UA (mg/dL)*	5 ± 0.3	5.7 ± 0.6	4.6 ± 0.3	4.5 ± 0.3	0.04	0.4	0.3
*CK (U/L)*	841 ± 136	708 ± 212	830 ± 85	650 ± 141	0.8	0.2	0.8

a *TN, thermoneutral; HS, heat stress; C, control; N, noni*.

b *E, environment; D, diet; ExD, interaction between environment and diet*.

c *Glc, glucose; chol, cholesterol; TG, triglycerides; LDH, lactate dehydrogenase; UA, uric. acid; CK, creatine kinase*.

### Noni supplementation modulates the hypothalamic expression of HSPs and HSFs

**Figure 4:**
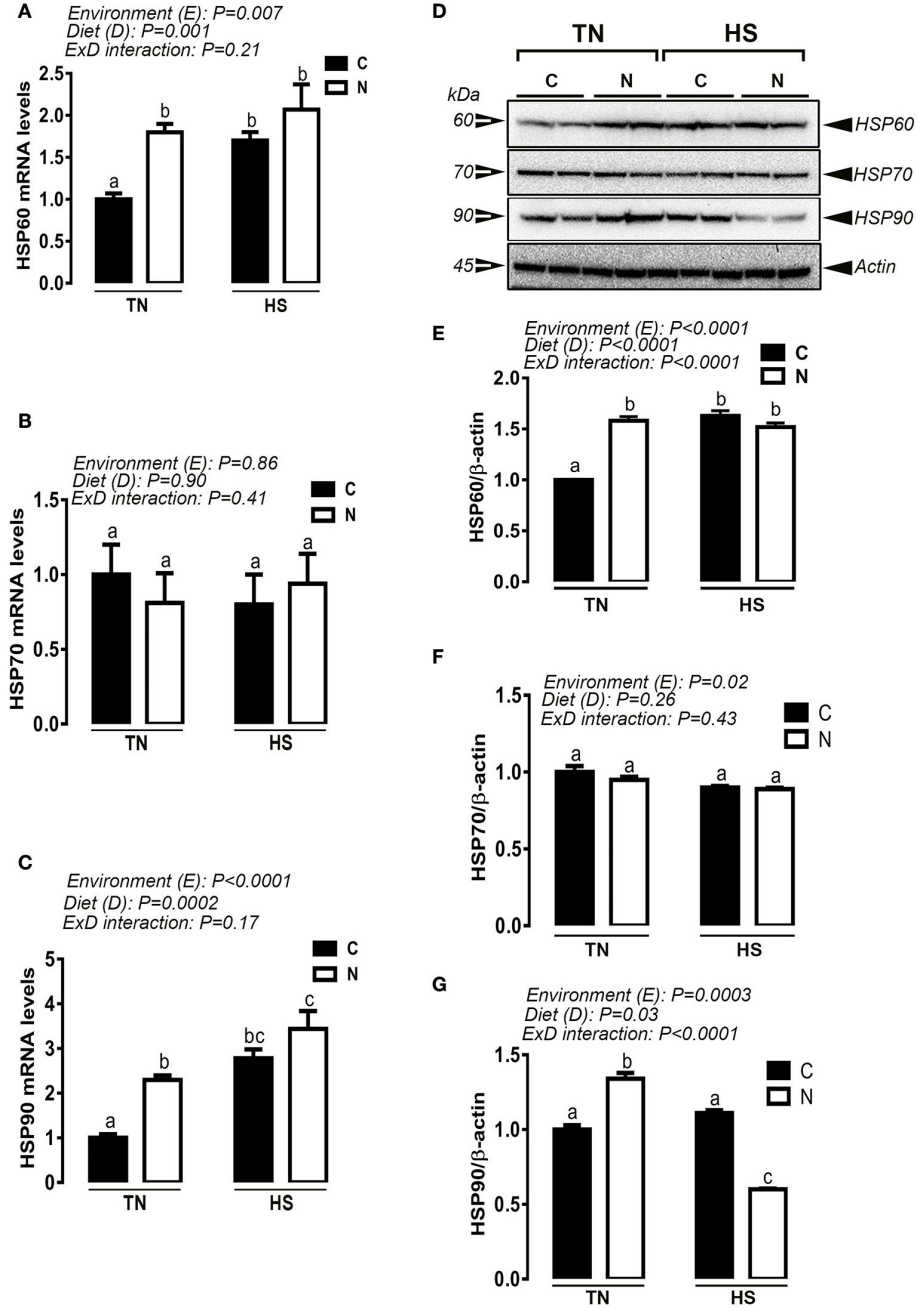
Effect of Noni supplementation on the hypothalamic expression of HSPs in acute heat-stressed (2 h) broilers. Relative mRNA expression of HSP60 **(A)**, HSP70 **(B)**, and HSP90 **(C)** was determined by qPCR. Protein levels of HSP60, HSP70, and HSP90 were assessed by Western blot **(D)** and presented as normalized ratio to the housekeeping protein β-actin **(E–G)**. Data are presented as mean ± SEM (*n* = 6/group). Different letters indicate significant difference at *P* < 0.05. Western blotting figure is representative **(A)** or three replicates **(E–G)**.

**Figure 5:**
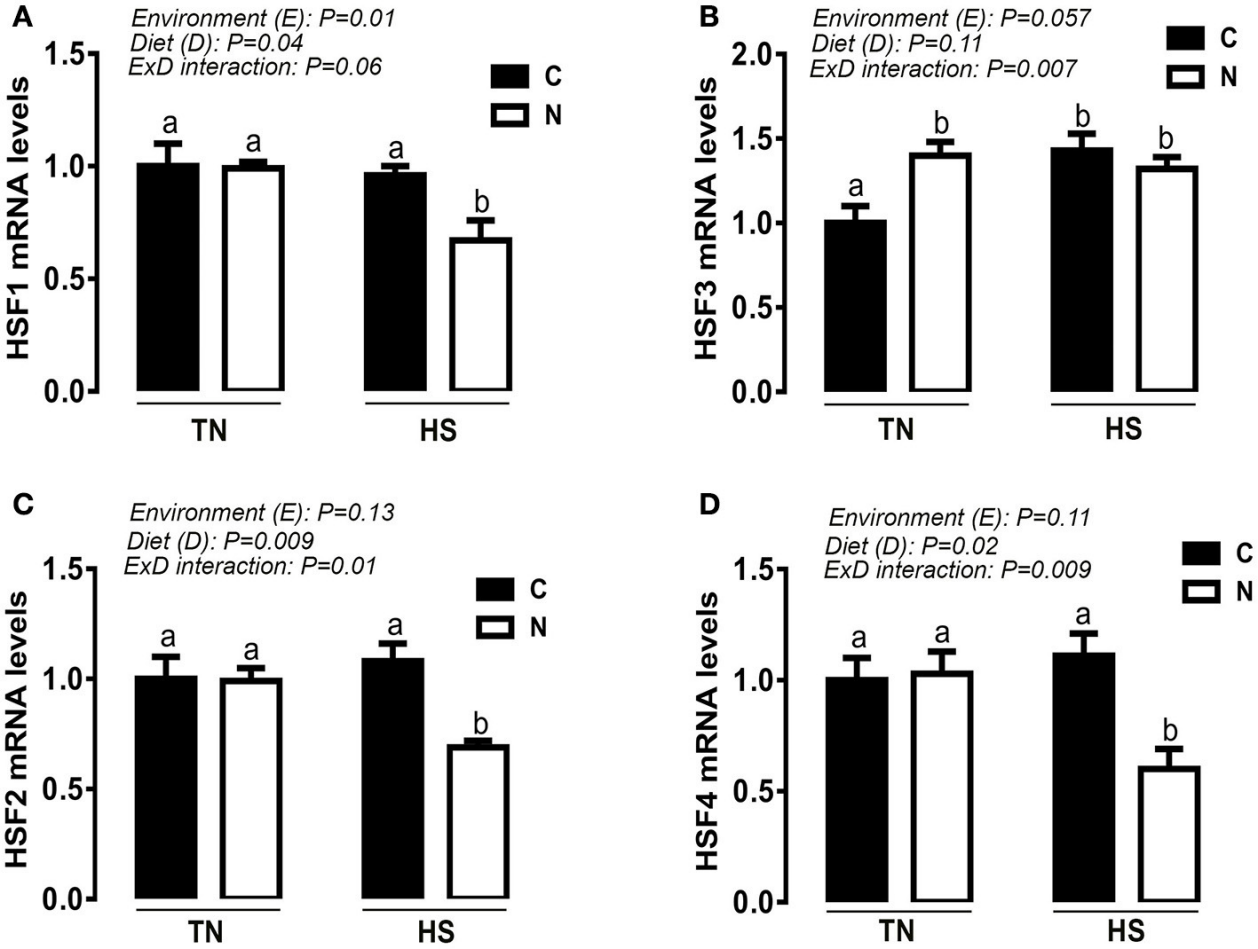
Effect of Noni supplementation on the hypothalamic expression of HSFs in acute heat-stressed (2 h) broilers. Relative expression of HSF1 **(A)**, HSF3 **(B)**, HSF2 **(C)**, and HSF4 **(D)** was determined by qPCR. Data are presented as mean ± SEM (*n* = 6/group). Different letters indicate significant difference at *P* < 0.05.

Under TN condition and prior to HS challenge, Noni supplementation significantly up-regulated the hypothalamic expression of HSP60 and HSP90 at mRNA and protein levels (e.g., Figures 4A,C,D). However, under acute HS condition, Noni significantly down regulated the hypothalamic expression of HSP90 only at protein but not at mRNA levels (Figures 4C,G). HSP60 mRNA abundance and protein levels remained unchanged between Noni and the control diet during HS (Figures 4A,E). The hypothalamic expression of HSP70 (mRNA and protein) did not differ between all the experimental groups (Figures 4B,F). Similarly, Noni significantly up regulated the hypothalamic expression of HSF3 gene in the TN group, and down regulated the expression of HSF1, 2, and 4 in the HS group (Figures 5A–D).

### Noni supplementation modulates the hypothalamic expression of feeding-related neuropeptides

**Figure 6:**
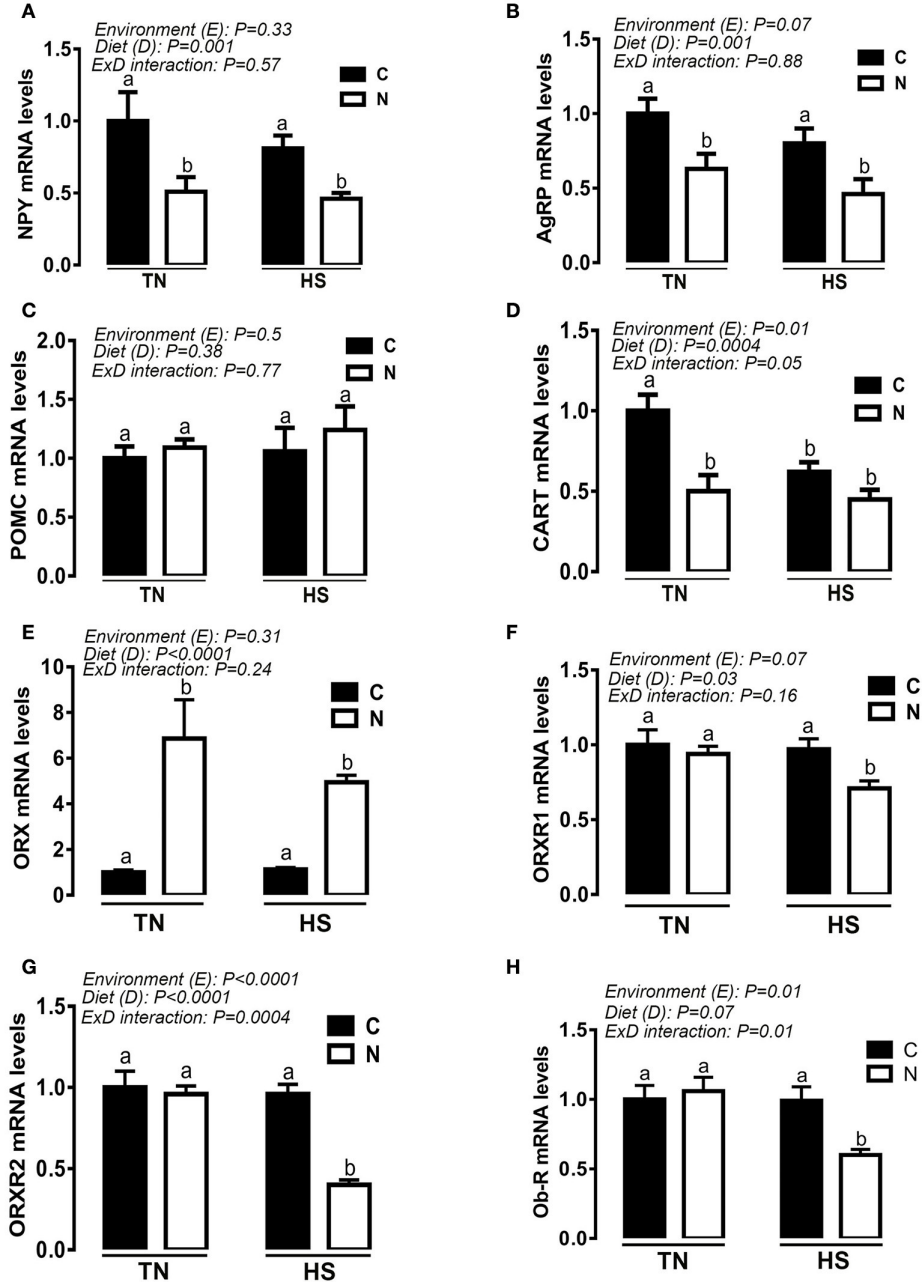
Effect of Noni supplementation on the hypothalamic expression of (an)orexigenic neuropeptides in acute heat-stressed (2 h) broilers. Relative expression of NPY **(A)**, AgRP **(B)**, POMC **(C)**, CART **(D)**, ORX **(E)**, ORXR1 **(F)**, ORXR2 **(G)**, and OB-R **(H)** mRNA was determined by qPCR. Data are presented as mean ± SEM (*n* = 6/group). Different letters indicate significant difference at *P* < 0.05.

Under TN conditions and prior to HS exposure, Noni supplementation significantly down regulated the hypothalamic expression of NPY, AgRP, and CART (Figures 6A,B,D) and up regulated that of ORX (Figure 6E). During the acute HS challenge, Noni did not affect the hypothalamic expression of CART (Figure 6D), but it did significantly decreased the mRNA abundances of NPY, AgRP, ORXR1, ORXR2, and Ob-R (Figures 6A,B,F–H), and increased that of ORX (Figure 6E). The hypothalamic expression of POMC gene did not differ between all the experimental groups (Figure 6C).

### Noni supplementation modulates the hypothalamic expression of AMPK-mTOR pathway

**Figure 7:**
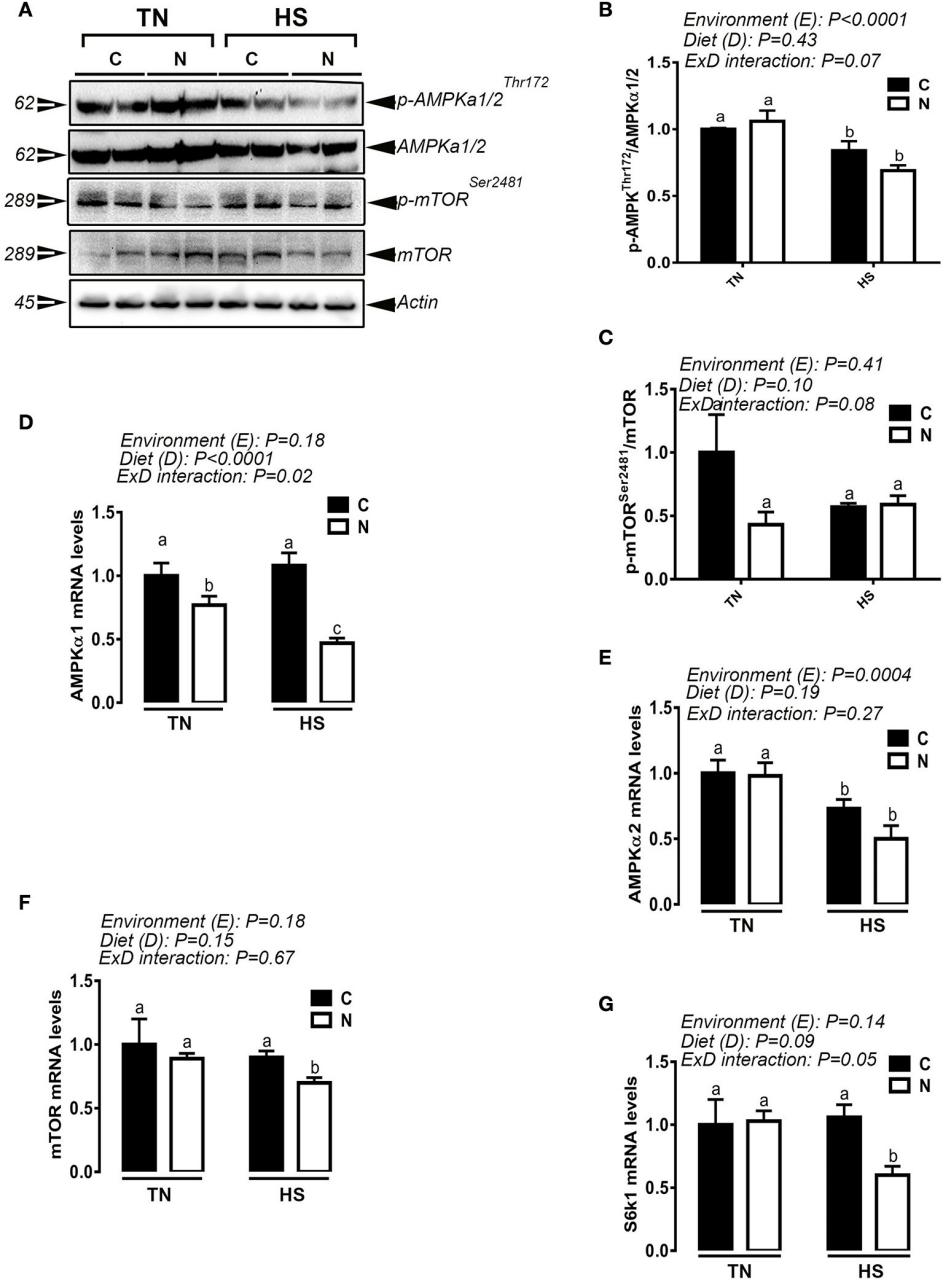
Effect of Noni supplementation on the hypothalamic expression and activation of AMPK-mTOR pathway in acute heat-stressed (2 h) broilers. Phosphorylated and pan levels of AMPKα1/2 and mTOR were determined using Western blot **(A)** and their relative expression was presented as normalized ratio of phosphorylated/total target protein **(B,C)**. Relative abundance of AMPKα1 **(D)**, AMPKα2 **(E)**, mTOR **(F)**, and S6K1 **(G)** mRNA was measured by qPCR. Data are presented as mean ± SEM (*n* = 6/group). Different letters indicate significant difference at *P* < 0.05. Western blotting figure is representative **(A)** or three replicates **(B–C)**.

Noni diet supplementation significantly decreased the phosphorylated levels of hypothalamic AMPKα1/2 at Thr172 in heat-stressed broiler and phospho-mTOR at Ser2481 in chickens maintained under both TN and HS conditions (Figure 7A). These changes were accompanied with a significant down regulation of the hypothalamic expression of AMPKα1, AMPKα2, mTOR, and S6K1 genes (Figures 7C–G).

## Discussion

Understanding acute heat shock paradigm, responses, and consequences is relevant for agriculture economics as well as for animal well-being and production sustainability. As depression in voluntary feed consumption is one of the prominent physiological responses of broilers to HS, we sought, in the present study, to mechanistically determine the effect of Noni on feeding behavior in broilers maintained under TN conditions or exposed to acute HS. As expected and in agreement with previous studies (Cooper and Washburn, 1998; Napper et al., 2015), acute HS increased the core BT and reduced feed intake in broilers. Interestingly, Noni supplementation delayed and reduced the increased BT-induced by HS. Although the molecular determinant are still unknown at this time, it is conceivable that Noni might modulate the thermoregulatory pathways to maintain BT homeostasis. It is possible that Noni might affect the expression of cutaneous, visceral, or central thermal receptors and increased blood flow and heat loss to the environment resulting in lower BT. It is also probable that Noni might affect the preoptic area integrity to influence warm-sensitive neurons and thereby drive thermogenic thermal effectors. As HSP90/HSF1 and leptin receptor are thought to play a fundamental role in thermal homeostasis (Nicholls et al., 2009; Kaiyala et al., 2016), the down regulation of their expression in our study indicate that Noni may reduce BT via modulation of HSP90 and/or leptin pathway.

Noni diet supplementation did not affect feed intake in our TN and HS experimental conditions. Previous studies reported that Noni supplementation improve body weight gain, FCR, and feed efficiency in indigenous (Nicobari fowl), broilers, and Japanese quails (Sunder et al., 2011, 2013). The apparent discrepancies between the results of our and previous studies might be related to experimental conditions including age/strain of chickens, duration and severity of heat stress, diet composition and levels of Noni incorporation, duration of Noni supplementation, and source of Noni extracts. For instance, Elfawati (2007) has shown an increase in appetite and feed intake using 7.5% Noni extract (Elfawati, 2007), however, Sunder and co-workers (Sunder and Kundu, 2015) reported a decrease in feed consumption using Noni juice (5 mL/bird/day). The same group (Sunder et al., 2013) reported that daily supplementation of Noni crude fruit extract at 5% enhanced the body weight gain and egg production in Japanese quails. In our study, we used 0.2% Noni fruit extract, the commonly incorporated levels by poultry producers in tropical regions. One additional factor that might be involved in the difference observed between the present and previous studies, is the environmental humidity levels. Indeed, the relative humidity was around 20% in our experimental condition compared to an average of 60–65% and even higher in tropical areas.

Selection and intake of diet depend not only on the available feed resource, but also on the feeding behavior of the animal. In attempt to better understand the effect of Noni on feeding behavior of broilers exposed to acute HS, we measured time spent eating and feeding frequency. We found that time spent eating, but not the number of access to the feeders, was reduced in heat-stressed chickens (in both control- and Noni-fed groups) compared to their TN counterparts which might explain the observed reduction in feed intake. The similar feed frequency observed between all experimental groups might due to social imitation and/or capacity of empathy (Zentall, 2004; Edgar et al., 2011). As expected, control diet-fed chickens exhibited higher drinking frequency and spent more time drinking when exposed to acute HS compared to those maintained under TN conditions. Noni supplementation, however, did not affect drinking behavior in chickens maintained under both environmental conditions (TN and HS). Interestingly, both control diet- and Noni-fed chickens manifested higher activity when they are exposed to acute HS compared to TN condition. This result is not surprising because acute heat-stressed chickens cannot lose heat through radiation and/or convection due to hot surrounding surfaces and lack of moving air. Instead, acute heat-stressed chickens adopted the conduction way by moving from one surface to another (toward the wall or on the litter) to lose heat; however, as these surfaces soon assume a temperature close to that of their body, chickens needed to move more frequently resulting in higher activity.

As feed intake is tightly controlled by the hypothalamic satiety and hunger centers (Sawchenko, 1998), we next determined the effect of Noni on the hypothalamic expression of feeding-related neuropeptides. HS alters the hypothalamic expression of CART, ORXR2, and Ob-R genes which may further explain and support the observed reduction in feed intake. Intriguingly, Noni supplementation increased the hypothalamic mRNA levels of orexin (ORX) in both TN and HS chickens, and decreased the mRNA abundance of orexin receptor 1/2 (ORXR1/2) and leptin receptor (OB-R) in heat-stressed broilers. Although orexin is well known as appetite- and feed intake-enhancing neuropeptide in mammals (Sakurai et al., 1998), its role is still speculative in avian species. In fact, Furuse's group have shown that intracerebroventricular (ICV) administration of orexin did not affect feeding behavior in neonate chicks (Furuse et al., 1999). Recently, our group made a breakthrough by identifying that orexin is expressed in chicken muscle and liver and it is responsive to oxidative and HS (Lassiter et al., 2015; Greene et al., 2016). Additionally, emerging evidence from mammalian studies identified orexin as a stress modulator via its interaction with the hypothalamic-pituitary-adrenal (HPA) axis (Bonnavion et al., 2015). Since Noni is known as an antioxidant and an anti-stressor, our results combined with previous mammalian studies suggest that Noni-orexin system interaction may play a key role in the central HS response in chickens. However, further mechanistic studies are warranted to define the mechanism(s) by which Noni regulates hypothalamic orexin expression and the role of Noni and orexin in HS response. The increased concentrations of circulating corticosterone by Noni in the present study supports our above mentioned hypothesis. Although there is still a long-standing debate over whether corticosterone is harmful or protective, it has been reported that corticosterone release during acute stress-induced activation of the HPA axis helps the body to overcome the negative effects of stress stimuli (Munck et al., 1984). Furthermore, it has been shown that orexin-producing neurons in the lateral hypothalamus regulate corticosterone release and a variety of physiological hallmarks of the stress response (Bonnavion et al., 2015), supporting again the hypothesis that Noni-mediating orexin-HPA axis may play a role in HS response in chickens. In continuation with the aforementioned changes, we found that HS differentially regulated the hypothalamic expression of HSPs, i.e., upregulation of HSP60 and HSP90 as well as their transcription factor HSF3 in chickens fed with control diet. However, HSP70 remained unchanged between all experimental groups. Noni supplementation seemed to induce HSP60 and HSP90 gene expression only in chickens maintained under TN conditions and to decrease the protein levels of HSP90 in heat-stressed chickens. As mentioned above, this reduction in HSP90 expression might mediate the effect of Noni in reducing BT-induced by HS (Nicholls et al., 2009). The absence of correlation between HSP90 mRNA and protein abundances indicated that Noni might regulate HSP90 at translational and post-translational levels as previously reported (Mollapour and Neckers, 2012). Despite the extensive studies in mammals, ample literature and scientific evidence much remains to be understood about the role of avian HSPs in normal and stress conditions. Owing to the gaps in avian HSP biology combined with their pleiotropic functions in mammals (housekeeping function, folding/unfolding, assembling/disassembling, intracellular transport and trafficking, protein degradation, cell signaling, etc.), interpretation and conclusions from our results are limited at this time point.

As HS alters the cellular energy homeostasis, and in order to gain better insights in the signaling cascades employed by Noni in a such condition, we determined the expression levels of the hypothalamic AMPK [the master energy sensor (Cantó et al., 2009)] and mTOR [the master nutrient sensor (Laplante and Sabatini, 2012)]. Although AMPK and mTOR have been recently shown to be expressed in the hypothalamus and regulate feed intake in mammals (Cota et al., 2006; Claret et al., 2007), our data indicated that this might not be the case in our experimental conditions as their expression did not differ between TN- and HS-chickens. Interestingly, Noni supplementation decreased the phosphorylated levels of hypothalamic AMPKα1/2 at Thr172 and mTOR at Ser2481 site in heat-stressed chickens suggesting that Noni might modulate this pathway to mitigate heat exposure-induced metabolic stress (Kishton et al., 2016).

In summary, although Noni did not alleviate reduction in feed intake in heat-stressed broilers, it did modulate the expression of hypothalamic stress- and metabolic-related markers, and this merits further in depth investigation.

## Author contributions

HR-S: performs the experiments; LE and TP: measure T3 and T4 concentrations; SD, WB, and AD: purchases reagents; SD: design the experiment; SD: writes the manuscript.

### Conflict of interest statement

The authors declare that the research was conducted in the absence of any commercial or financial relationships that could be construed as a potential conflict of interest.
